# Equal antipyretic effectiveness of oral and rectal acetaminophen: a randomized controlled trial [ISRCTN11886401]

**DOI:** 10.1186/1471-2431-5-35

**Published:** 2005-09-06

**Authors:** Mona Nabulsi, Hala Tamim, Ramzi Sabra, Ziyad Mahfoud, Shadi Malaeb, Hadi Fakih, Mohammad Mikati

**Affiliations:** 1 Department of Pediatrics, American University of Beirut Medical Center, Beirut, Lebanon; 2 Department of Epidemiology and Population Health, Faculty of Health Sciences, American University of Beirut, Beirut, Lebanon; 3 Department of Pharmacology and Therapeutics, Faculty of Medicine, American University of Beirut, Beirut, Lebanon; 4 Department of Pediatrics, Middle East Hospital, Beirut, Lebanon

## Abstract

**Background:**

The antipyretic effectiveness of rectal versus oral acetaminophen is not well established. This study is designed to compare the antipyretic effectiveness of two rectal acetaminophen doses (15 mg/kg) and (35 mg/kg), to the standard oral dose of 15 mg/kg.

**Methods:**

This is a randomized, double-dummy, double-blind study of 51 febrile children, receiving one of three regimens of a single acetaminophen dose: 15 mg/kg orally, 15 mg/kg rectally, or 35 mg/kg rectally. Rectal temperature was monitored at baseline and hourly for a total of six hours. The primary outcome of the study, time to maximum antipyresis, and the secondary outcome of time to temperature reduction by at least 1°C were analyzed by one-way ANOVA. Two-way ANOVA with repeated measures over time was used to compare the secondary outcome: change in temperature from baseline at times1, 2, 3, 4, 5, and 6 hours among the three groups. Intent-to-treat analysis was planned.

**Results:**

No significant differences were found among the three groups in the time to maximum antipyresis (overall mean = 3.6 hours; 95% CI: 3.2–4.0), time to fever reduction by 1°C or the mean hourly temperature from baseline to 6 hours following dose administration. Hypothermia (temperature < 36.5°C) occurred in 11(21.6%) subjects, with the highest proportion being in the rectal high-dose group.

**Conclusion:**

Standard (15 mg/kg) oral, (15 mg/kg) rectal, and high-dose (35 mg/kg) rectal acetaminophen have similar antipyretic effectiveness.

## Background

Parents of febrile children often conceive fever as a disease that requires treatment, rather than being a symptom or a sign of illness. In their anxious quest to treat fever, parents suffering from "fever phobia" may end up unintentionally overdosing their children with different antipyretics, or with different preparations of the same antipyretic [1-3]. Acetaminophen, in its various preparations, is a widely used drug because of its established analgesic and antipyretic effects. Whereas the analgesic efficacy achieved with standard (10–20 mg/kg) oral, standard rectal (10–20 mg/kg), and high rectal (40–45 mg/kg) acetaminophen doses have been well investigated [4-7], the comparative antipyretic effects of oral and rectal acetaminophen is not well studied. Parents, as well as physicians, use the standard dose (10–20 mg/kg) of oral and rectal acetaminophen preparations interchangeably to treat fever in children, assuming they have equal antipyretic effects. However, although the evidence for rapid absorption (within 30–60 minutes) and the pharmacokinetics of a single acetaminophen oral dose is well-established [8,9], the pharmacokinetics of a single rectal dose reveal the absorption to be erratic and prolonged, varying with the suppository size, composition of its base, rate of dissolution, position in the rectum, and the rectal contents [5]. Moreover, an increasing body of evidence indicates that the rectal acetaminophen dose of 10–15 mg/kg fails to achieve antipyretic serum levels of 10–20 μg/ml. Indeed, a rectal acetaminophen dose of 30–45 mg/kg is needed to achieve antipyretic serum levels in that range [5,7,10-12].

To our knowledge, only three randomized controlled trials had previously investigated the antipyretic effects of rectal acetaminophen in comparison to the oral one, with contradictory results [13-15]. Whereas Leary et al. found oral paracetamol to be superior to the rectal preparation in reducing the temperature of febrile children [13], both Vernon et al [15] and Scolnik et al [14] found no difference in the antipyretic responses of oral and rectal acetaminophen. However, Vernon's study was unblinded and lacked placebo control, and compared the standard doses of 15–20 mg/kg of oral and rectal acetaminophen only. Scolnik's study, also lacking blinding and placebo control, was further limited by the fact that it assessed antipyresis during the first three hours after drug administration, a time during which maximum antipyresis of rectal acetaminophen may not have occurred.

In view of the conflicting results in the literature, we conducted this study to compare the antipyretic effectiveness of two different rectal doses of acetaminophen: 15 mg/kg and 35 mg/kg to that of a standard oral dose of 15 mg/kg, over a six-hour period to allow detection of late antipyresis that may occur with rectal acetaminophen. The results of this study will provide further evidence on the comparative antipyretic effects of different doses of rectal acetaminophen versus the standard oral one.

## Methods

### Setting

This study was conducted between November 2000 and September 2002, in the paediatric inpatient services of two hospitals in Beirut: the American University of Beirut Medical Center (AUBMC), which is a tertiary care facility, and the Middle East Hospital (MEH), a secondary care facility. The Institutional Review Board and the Ethics Committee at the American University of Beirut, as well as the Board of the Middle East Hospital, approved this study. Written informed consent was obtained from all parents as well as the oral consent of children aged 10 years or more.

### Subjects

Subjects approached for enrolment in the study were febrile inpatients whose ages were between 6 months and 13 years, and whose rectal temperature was ≥ 38.5°C. A wide age range was permitted to enhance recruitment, since the antipyretic effect of acetaminophen does not vary with age [9]. Exclusion criteria included any of the following conditions: acute or chronic gastroenteritis, vomiting, any medical or surgical condition that precluded oral or rectal drug administration, acute or chronic hepatic disease, rectal bleeding, malabsorption syndromes, acute or chronic renal disease with the exception of urinary tract infection, chronic metabolic disease, bleeding disorders, chronic neurological disease that may affect central thermoregulation, cancer, immune suppression, sepsis, critical medical status, or known allergy to acetaminophen. Children with concurrent or previous intake of antibiotics were not excluded. All antipyretics were stopped for 8 hours prior to the initiation of the study.

### Study design

This is a randomized, double blind, and double dummy design clinical trial. Subjects were randomized according to a computer-generated, random-number list that was kept with the hospital pharmacist until the end of the study, into one of three treatment groups: standard oral acetaminophen dose (15 mg/kg) and rectal placebo suppositories; standard rectal acetaminophen dose (15 mg/kg) and oral placebo; high-dose rectal acetaminophen (35 mg/kg) and oral placebo. The allocation sequence was generated by one of the co-investigators (HT) who was not involved in subject enrolment or outcome assessment. The pharmacist who prepared the study medications was aware of subjects' treatment allocation, whereas subjects, parents, nurses, treating physicians, research assistant responsible for subject enrolment, data analyst (co-investigator ZM) and investigators were all blinded to the assignment of the patients.

### Study medications

The drugs used in this study: acetaminophen and its placebo were supplied by Julphar (Gulf Pharmaceutical Industries, United Arab Emirates). The oral acetaminophen used was a 250 mg acetaminophen/5 ml suspension (Adol, Julphar), while the placebo was a suspension with a similar colour and exipient to Adol. The suppositories (Adol) came in three sizes: 125 mg, 250 mg, and 500 mg. The suppository base is lipophyllic and consists of semi-synthetic glycerides (1140 mg of saturated fatty acids from C8 to C18). Placebo suppositories consisted of the same base, and came in similar colour, shape, and sizes.

### Study procedure

After obtaining the approval of the treating physician, the parent(s) of the eligible child was approached for interview and enrolment. During the interview, a trained research assistant administered a structured questionnaire designed to collect information on the following variables: diagnosis, previous or concurrent antibiotics, antipyretic intake, fever duration, gender, and date of birth. The purpose and procedure of the trial were fully explained to the family, and written parental consent, as well as oral consent of the subject when older than ten years of age were obtained. Children enrolled in the study were then assigned a random number as mentioned previously.

Baseline rectal temperature was recorded using a portable thermistor with single-use disposable probe covers (Sure Temp 679, Welch Allyn). One thermometer was used for the whole duration of the study in each hospital. Investigational drugs were prepared by the pharmacist who was aware of subjects' treatment allocation as follows: the oral group would receive oral acetaminophen at a dose of 15 mg/kg through a syringe, followed by placebo rectal suppositories which were estimated assuming a rectal acetaminophen dose of 35 mg/kg; the second group would receive oral placebo at a volume similar to the volume obtained if oral acetaminophen were to be given at a dose of 15 mg/kg, and rectal suppositories consisting of 15 mg/kg acetaminophen and 20 mg/kg placebo; the third group would receive oral placebo, and rectal acetaminophen suppositories at 35 mg/kg dose. In order to avoid cutting suppositories, rectal acetaminophen dose was rounded to the suppository size nearest to the calculated dose. More than one suppository could be used to achieve the desired rectal dose. The patient's nurse, who was blinded to the treatment allocation, administered all drugs and checked suppository retention for 30 minutes following administration and at hourly intervals for the duration of the study. Rectal temperatures were subsequently recorded at 1, 2, 3, 4, 5, and 6 hours from baseline.

### Statistical analyses

The primary outcome was time to maximum antipyresis following administration of a single dose of acetaminophen. Secondary outcomes: included time to fever reduction by at least 1°C, the temperatures at one, two, three, four, five, and six hours from administration and possible side effects such as hypothermia defined as a rectal temperature < 36.5°C.

For sample size calculation, we considered a one-hour difference in the average time to reach maximum antipyresis between any of two treatment groups to be a clinically significant outcome. Using this one-hour difference to maximum antipyresis, a standard deviation of one hour, 80% power, and alpha of 0.05, the calculated sample size was 48 subjects, 16 in each group.

We used the Chi square test to study the association between categorical variables and treatment groups, and one-way ANOVA to investigate the relationship between continuous variables and treatment groups. Two-way ANOVA with repeated measures over time was used to compare the changes in temperature from baseline at each time (t = 1, 2, ..., 6), among the three groups. Intent-to-treat analysis was planned with statistical significance set at P < 0.05.

## Results

### Baseline characteristics

**Figure 1:**
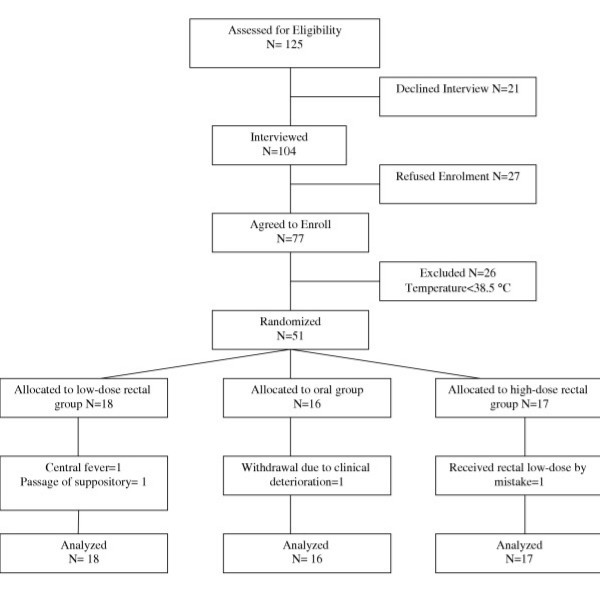
Flow diagram of the subjects' progress through the study.

Between November 2000 and September 2002, 125 parents were approached for interview and questionnaire administration. The progress of these subjects through the study is shown in Figure 1. There were no differences in the baseline characteristics of the patients who completed the study and those who did not. The study was completed with 51 subjects: 16 in the oral group, 18 in the rectal standard-dose group, and 17 in the rectal high-dose group. Their mean (SD) age was 3.9(3.0) years, with an age range of 6 months-13.1 years. The median duration of fever was 3.0 days, with a range of 0.5–101.0 days. Three patients had prolonged fever ranging from 3 weeks to 3 months that were later diagnosed to be due to juvenile rheumatoid arthritis, central fever, and viral etiology. These patients were analyzed in their groups since intent to treat analysis was planned. There were 29 (56.9%) males, and 35 (68.6%) subjects were receiving at least one antibiotic when entered into the study.

The characteristics of the three groups are shown in Table 1. There were no significant differences with respect to sex, age, underlying basic disease causing fever, duration of fever, previous antipyretic use, or concurrent antibiotic administration. In addition, there were no differences in the characteristics or treatment allocation of the patients recruited from AUBMC (39), and those recruited from MEH (12). The baseline temperature was similar in the three groups with a mean (SD) of 39.2 (0.7)°C. As for the rectal acetaminophen doses, the means (SD) and ranges were: 14.1 (2.3) mg/kg and 10.7–18.5 mg/kg for the standard rectal dose; and 31.7 (6.7) mg/kg, and 12.5–43.4 mg/kg for the rectal high-dose respectively. One subject who was allocated to the rectal high-dose group received a low rectal dose by mistake. Excluding the dose received by this patient, the mean (SD) and range for the high-dose rectal group becomes 33.0(4.7) mg/kg and 27.2–43.4 mg/kg respectively. Since intent-to-treat analysis is planned, this subject was analyzed in the high-dose group.

**Table 1:** Subject characteristics and main outcome.

	**TOTAL**	**RECTAL (15 MG/KG)**	**ORAL (15 MG/KG)**	**RECTAL (35 MG/KG)**
**Total**	51	18	16	17

**Male Gender**				
N (%)	29 (56.9)	11	8	10

**Age (years):**				
Mean (SD)	4.1 (3.5)	3.8 (2.8)	4.0 (3.6)	3.5 (3.0)
Range	0.5–13.1	0.6–13.1	0.5–10.2	0.5–12.4

**Diagnosis:¶ N (%)**				
Pneumonia	10 (19.6)	3	2	5
UTI	7 (13.7)	2	4	1
Virus	17 (33.3)	6	4	7
Bacteremia	5 (9.8)	2	2	1
Others	36 (70.5)	15	12	9

**Previous antipyretic **				
N (%)				
Acetaminophen	40(78.4)	15	14	11
Ibuprofen	8(15.6)	2	2	4

**Antibiotic intake **				
**N (%)**	35 (68.6)	16	8	11

**Acetaminophen Dose (mg/kg):**				
Mean (SD) **§**		14.1(2.3)	15.0(0.0)	31.7(6.7)
Range		10.7–18.5	15.0–15.0	12.5–43.4

**Duration of fever (days):**				
Median	3.0	3	1.3	5
Range	0.5–101.0	1.0–90.0	0.5–15.0	1.0–101.0

**Baseline temperature**				
Mean (SD)	39.2 (0.7)	39.1 (0.9)	39.3 (0.6)	39.1 (0.6)

**Time to max AP in hr:**				
**Mean**	3.6	3.3	3.6	3.9
**95% CI**	3.2–4.0	2.4–4.2	2.8–4.3	3.3–4.6

§ P = 0.000 (ANOVA); ¶ Percentages do not add up to 100 since more than one diagnosis is entered for some subjects; max AP: maximum antipyresis; hr: hour.

### Primary outcome

Intent-to-treat analysis of the time to maximum antipyresis, the primary outcome measure of the study, revealed no significant differences among the three groups (P = 0.5). The overall mean (95% CI) was 3.3 (2.4–4.2) hours for the rectal low-dose group, 3.6 (2.8–4.3) hours for the oral group, and 3.9 (3.3–4.6) hours for the rectal high-dose group [Table 1]. Repeat analysis excluding the three patients with long duration of fever, and the only patient who received a low-dose rectal acetaminophen instead of his allocated high-rectal dose, revealed similar results to the intent-to-treat analysis. Therefore, analyses of both primary and secondary outcomes were kept as intent-to-treat.

### Secondary outcomes

The mean (95%CI) maximum decline in temperature was 1.6(1.3–2.0)°C in the rectal low-dose group, 1.7(1.2–2.2)°C in the oral group, and 2.0(1.4–2.5)°C in the rectal high-dose group (P = 0.5). The time to fever reduction by at least 1°C was similar among the three groups: mean (95% CI) of 2.4 (1.8–3.1) hours in the rectal low-dose, 3.5 (2.6–4.4) hours in the oral and 2.8 (2.1–3.6) hours in the rectal high-dose groups (P = 0.13). Two-way ANOVA with repeated measures over time did not reveal statistically significant differences in the changes in temperature from baseline at times 1, 2, ..., 6 hours among the three groups (P = 0.25).

As for side effects of the medications, hypothermia, defined as a body temperature below 36.5°C rectally, occurred in 11 (21.6%) subjects: 2(11.1%) with the rectal standard dose, 3(18.8%) in the oral group and 6(35.3%) with the rectal high-dose. These proportions however were not statistically significant (P = 0.2). The temperature range of hypothermic episodes was between 35.5°C and 36.4°C (mean 36.1°C).

There were three mortalities among our subjects, which were judged to be unrelated to the investigational drugs. The first one was a one-year-old male infant who died 10 days after enrolment from systemic Epstein-Barr viral infection. The second mortality occurred in a 12-year-old child who succumbed to bacterial endocarditis and myocardial abscesses two weeks following enrolment in the study, and the third patient was a six month old boy whose clinical status deteriorated four hours after enrolment, the time at which he was withdrawn from the study. This patient died 14 hours later from complicated respiratory infection, sepsis, and respiratory failure.

## Discussion

Acetaminophen is the most widely used antipyretic in paediatric medicine. Despite the well-established antipyretic effects of oral and rectal acetaminophen, controversy regarding the comparative antipyretic effectiveness of the two types of acetaminophen preparations is yet unresolved. Whereas some investigators have reported better antipyresis with oral acetaminophen [13], others have reported equal antipyretic effects [14,15]. Faced with this uncertainty, the use of either preparation is often influenced by the child's acceptance of the oral medication, his medical condition (presence of vomiting for example), and parental or physician preferences.

Our results reveal no difference in the antipyretic effectiveness among oral, rectal standard-dose, or rectal high-dose acetaminophen. The time to maximum antipyresis was not significantly different among the three doses or preparations of the drug, with an overall mean (SD) of 3.3(95%CI: 2.4–4.2) hours. In addition, the three regimens behaved similarly with respect to the maximum decline in temperature at any time during the six hours and the time to fever reduction by at least one degree. Our findings are in agreement with those of Vernon, et al [15] and Scolnik, et al [14], but different from those of Leary et al [13]. These differences may be attributed to the fact that in Leary's study, all outcome measurements were based on axillary temperatures, the reliability of which is uncertain [16].

This study is the fourth randomized controlled trial that compares the antipyretic effectiveness of oral and rectal acetaminophen, and the second one to investigate the differences in antipyresis between standard oral, standard rectal, and high-dose rectal acetaminophen. The strengths of this study, as compared to the previous ones, include the fact that the six-hour study duration permitted detection of any delayed antipyretic responses, if present. In addition, it was double-blinded with double-dummy technique. In contrast, the study of Scolnik et al [14] assessed antipyresis for the first three hours only, the time at which maximum antipyresis may not have occurred. In addition, it was neither blinded, nor placebo-controlled. Similarly, Vernon et al's study [15] lacked both blinding and placebo control, and compared the standard doses of 15–20 mg/kg of oral and rectal acetaminophen only. Finally, the main drawback of Leary et al's study [13] was their use of axillary temperature instead of the gold standard rectal measurements, which undermines the reliability of their results.

Our study is limited by the fact that it included inpatients only, the majority of which were on antibiotics and had previously received antipyretics. However, our findings can be generalized to febrile children who are treated as outpatients, since the antipyretic response to acetaminophen is not known to vary between inpatients and outpatients. In addition, and since our outcome of interest is "effectiveness" rather than "efficacy", we did not exclude subjects receiving antibiotics from enrolment nor subjects with prior intake of antipyretics. Antipyretics however were stopped for 8 hours prior to enrolment, the time at which a febrile subject may receive antipyretic treatment in "real clinical life". It may be argued that the antipyretic effects of the investigational drugs are confounded by antibiotic administration and previous antipyretic intake. However since this is a randomized clinical trial, we anticipated that the randomization process will dilute these effects by distributing these subjects equally among the three treatment groups. Indeed, the proportions of subjects receiving antibiotics and those with prior antipyretic intake were not significantly different among the three groups, suggesting adequate randomization. A weakness of this study is the inter-individual variability of the acetaminophen dose in the rectal high-dose group which ranged between 27.2 and 43.4 mg/kg, after exclusion of the subject who received a low dose by mistake. It is possible that the lower acetaminophen doses in this range may have attenuated the mean antipyretic effect of this group resulting in similar antipyretic responses among the three different groups. This problem however is difficult to avoid with rectal administration and is frequently encountered in real clinical life. We cannot therefore eliminate the possibility of some imprecision of the results in the rectal high-dose group due to dosage variability.

It is interesting to note that one fifth of our patients developed hypothermia during the study interval, a finding that has not been previously reported. Though the differences in the proportions of patients with hypothermia among the three groups did not achieve statistical significance, the rectal high-dose group tended to have a higher proportion with hypothermia. This observation needs to be further investigated with a larger sample size, since our study was not powered to detect whether hypothermia is more common in one group as compared to the others.

## Conclusion

In conclusion, oral and rectal acetaminophen preparations seem to have equal antipyretic effectiveness which is in line with earlier studies. There is no evidence to support the belief that rectal suppositories, whether prescribed in the standard dose of 15 mg/kg, or in the high dose of 30–40 mg/kg, are superior to oral acetaminophen in terms of rapidity of action, or in the extent of temperature reduction. Though the oral route may be preferred because of its predictable rapid absorption, the rectal route seems to be a good and equally effective alternative in special circumstances like vomiting, or conditions preventing oral administration. High-dose rectal acetaminophen should be used with caution, since it may result in hypothermia, a finding that deserves further exploration in the future. Physicians should educate parents about fever being a benign symptom of illness, rather than a disease in itself. While it is desirable to treat fever in children, parents need to be aware that fever per se is not a usual cause of mortality in a child, while acetaminophen overdose can be [1-3].

## Competing interests

The author(s) declare that they have no competing interests.

## Authors' contributions

MN prepared grant submission in relation to this study, contributed to the design, data acquisition, analysis and interpretation, drafting, revision and final approval of the manuscript. HT contributed to the design, data analysis and interpretation, drafting, revision and final approval of the manuscript. RS participated in grant submission, design, revision and final approval of the manuscript. ZM contributed to statistical analysis, revision and final approval of the manuscript. SM and HD contributed substantially to data acquisition, drafting and final approval, MM participated in grant submission, drafting, revision and final approval of the manuscript.

## Pre-publication history

The pre-publication history for this paper can be accessed here:


